# Apoptotic mesenchymal stromal cells support osteoclastogenesis while inhibiting multinucleated giant cells formation in vitro

**DOI:** 10.1038/s41598-021-91258-4

**Published:** 2021-06-09

**Authors:** Paul Humbert, Meadhbh Á. Brennan, Julien De Lima, Régis Brion, Annie Adrait, Céline Charrier, Bénédicte Brulin, Valérie Trichet, Yohann Couté, Frédéric Blanchard, Pierre Layrolle

**Affiliations:** 1 UMR 1238, Phy-OS, Bone Sarcoma and Remodeling of Calcified Tissues, School of Medicine, University of Nantes, INSERM, 44000 Nantes, France; 2 Regenerative Medicine Institute, School of Medicine, and Bioengineering Department, School of Engineering, National University of Ireland, Galway, H91 TK33 Ireland; 3 CHU Nantes, 44000 Nantes, France; 4 Université Grenoble Alpes, CEA, INSERM, IRIG, BGE, 38000 Grenoble, France

**Keywords:** Bone, Stem-cell research

## Abstract

In bone regeneration induced by the combination of mesenchymal stromal cells (MSCs) and calcium-phosphate (CaP) materials, osteoclasts emerge as a pivotal cell linking inflammation and bone formation. Favorable outcomes are observed despite short-term engraftments of implanted MSCs, highlighting their major paracrine function and the possible implication of cell death in modulating their secretions. In this work, we focused on the communication from MSCs towards osteoclasts-like cells in vitro. MSCs seeded on a CaP biomaterial or undergoing induced apoptosis produced a conditioned media favoring the development of osteoclasts from human CD14+ monocytes. On the contrary, MSCs’ apoptotic secretion inhibited the development of inflammatory multinucleated giant cells formed after IL-4 stimulation. Components of MSCs’ secretome before and after apoptotic stress were compared using mass spectrometry-based quantitative proteomics and a complementary immunoassay for major cytokines. CXCR-1 and CXCR-2 ligands, primarily IL-8/CXCL-8 but also the growth-regulated proteins CXCL-1, -2 or -3, were suggested as the major players of MSCs’ pro-osteoclastic effect. These findings support the hypothesis that osteoclasts are key players in bone regeneration and suggest that apoptosis plays an important role in MSCs’ effectiveness.

## Introduction

The combination of culture-expanded autologous mesenchymal stromal cells (MSCs) from bone marrow and calcium-phosphate (CaP) materials has been increasingly studied for bone regeneration therapies. It has proven efficacy in preclinical models^1^, in both ectopic and orthotopic sites, and in clinical trials for the treatment of non-unions^2,3^ as well as maxilla-facial defects^4^. The ongoing ORTHOUNION European project aims to evaluate the efficacy and the cost effectiveness of this cell therapy in comparison to autologous bone grafting^5^. Despite promising results, and although the clinical use of autologous MSCs resolve many of the limitations of bone grafting, it also presents some major challenging issues. While less invasive than bone harvesting for autologous grafting, bone marrow aspiration remains a surgical procedure comprising risks. The variability between donors may alter the expansion efficiency and the clinical outcome. The use of living cells comes with substantial regulatory constraints^6^ and the cost efficiency of the procedure still remains to be evaluated. Therefore, a better understanding of the exact mechanism of osteoinduction by MSC-CaP therapies is essential to rationally design future cell-free approaches for bone regeneration and improve further patients care.

Both the biomaterial properties (including porosity, surface structure, chemical composition)^7^ and the cells’ characteristics (including tissue of origin, passage, dose)^8,9^ are important in modulating the host response and directing the outcome of implantation. From our own experimental observations^10^, together with an in-depth review of the literature on this topic, we recently presented a hypothetical mechanism of bone formation whereby osteoclasts are key cells that turn the early inflammatory reaction towards a bone formatting cascade^11^. In successful bone forming conditions, several studies from other groups also reported a rapid clearence of implanted MSCs^12,13^, while early osteoclast formation directly on the biomaterial was reported^14^. We postulated that stressed MSCs on CaP materials can locally direct myeloid cell differentiation, i.e. favor the development of osteoclasts instead of multinucleated giant cells (MNGCs), typical of the foreign body reaction. Consistent with the plasticity of macrophages, two different phenotypes of MNGCs could favor either inflammation or wound healing^15^. However, osteoclasts are the physiological multinucleated cells of bone responsible for its resorption and, most importantly here, the major sources of coupling factors with osteoblast lineage cells^16^. While physiological remodeling happens on bone surfaces, we hypothesized that once formed on the surface of the biomaterial, osteoclast could also attract new skeletal stem cells and participate in their differentiation into bone forming osteoblasts while MNGCs would favor fibrosis. In vivo observations lead us to this potential two-step mechanism but it remains to be formerly proven and explore in depth.

MSCs secrete a variety of immunomodulatory factors^17^, essential to attenuate initial host reaction against the biomaterial and avoid chronic inflammation. Death of implanted cells is mostly attributed to a lack of oxygen^18^ and nutrients^19^. These factors, and other stresses such as the inflammatory environment, the reaction to the biomaterial surface or the mechanical constrain, influence MSCs to such an extent that MSC pre-conditioning before implantation is being explored to enhance their therapeutic potential^20,21^. In addition, in vitro collection of MSCs’ secretions after stimulation could represent a valuable clinical tool. Apoptosis itself is a source of immunomodulatory signals that could be involved in MSC-based therapies^22^.

MSCs have been described as either supportive^23,24^ or suppressive^25,26^ towards osteoclastogenesis depending on the culture conditions and the cell source. In this study, we questioned the possibility that MSCs could trigger osteoclast formation to the detriment of MNGCs, as the first step of our hypothetical mechanism. In addition, identifying soluble mediators involved in this communication would be key to further decipher the bone formation process or develop new therapeutic approaches. To this end, the effect of MSCs’ secretion on osteoclasts and MNGCs was assessed in vitro*,* using primary human cells. Conditioned media (CM) from human bone marrow MSCs were initially collected after cell seeding on the biphasic calcium phosphate (BCP) material used in the ORTHOUNION clinical trial. This model was later simplified into a two-dimensional culture where apoptotic stress was induced by a staurosporine (STS) treatment. We explored the composition of the CMs by high-throughput proteomic analysis using bottom-up mass spectrometry (MS)-based analysis and multiplex immunoassay. The implication of major candidates was evaluated using neutralizing antibodies targeting cytokine and chemokine receptors on the osteoclast membrane.

## Methods

All cell culture manipulations were performed under sterile conditions. Cells were incubated in a humid atmosphere at 37 °C, 5% CO_2_.

## MSCs culture

Bone marrow MSCs from five healthy donors were obtained from the Institut für Klinische Transfusionsmedizin und Immungenetik of Ulm, Germany. The isolation and validation procedure of these cells following Good Manufacturing Practices (GMP) was previously described by Rojewski et al.^27^. Specific information on each donor cells used in the present study can be found as supplemental data in the previously cited article. Bone marrow was collected after written informed consent was obtained according to the Declaration of Helsinki and approved by the Ethics Committee of Ulm University (ethical approval numbers 21/10 and 24/11). Trilineage differentiation capacity (adipogenic, chondrogenic and osteogenic) and surface marker analysis (negative for CD3, CD34, CD45 and MHC cI, and positive for CD73, CD90, CD105 and MHC cII) ensured that isolated cells followed the minimal criteria for mesenchymal stem cells^28^. Karyotyping as well as microbial, mycoplasma and endotoxin testing were also performed as part of the GMP quality control. Donors are encoded as follow, their age and sex specified in brackets: APS 7554 (22, male), APS 7553 (22, male), APS 7537 (28, female), ALA 7543 (23, male) and APA 7535 (26, female). Cells were grown in αMEM (Gibco, 22571020) containing 1% Penicillin/Streptomycin mixture (P/S, Eurobio, CABPES010U) and 5% pooled human platelet lysate with heparin (PLP, 1 IU/ml of final media).

For 3D experiments, 400,000 cells were seeded in serum free media (αMEM, 1%P/S), either on 50 mg of MBCP+ or in 15 mL Falcon tubes and centrifuged (500 g, 5 min) to form spheroids. For experiments using staurosporine (STS, Santa Cruz, sc-3510), cells were seeded in T75 flasks and incubated until 80 to 90% confluence was reached. The treatment consisted of 4 h in 20 mL serum-free media containing 0.1 µM STS or without STS as a control (untreated, UNT). The flasks were then washed 3 times with phosphate buffer saline (PBS) to remove excess STS and 20 mL of fresh serum-free media was added. The supernatants were collected after 48 h, filtered at 0.22 µm, aliquoted and stored at − 20 °C until use.

### Osteoclasts and MNGCs differentiation

Circulating monocytes were isolated from concentrated peripheral blood of healthy individuals, provided by the Etablissement Français du Sang as leftovers of platelet donation. The blood was flushed out of the sorting system and diluted with PBS. This diluted solution was carefully deposited on an equivalent volume of Ficoll-Paque Premium (GE Healthcare) and centrifuged (800 g, 20 min). Peripheral blood mononuclear cells at the interface were recovered using a Pasteur pipette, wash 3 times with PBS and counted. CD14 positive cells were isolated using hCD14 MicroBeads (Miltenyi Biotec, 130-050-201) and LS Columns (Miltenyi Biotec, 130-042-401) according to the manufacturer’s instruction. CD14+ enriched cells were stored for up to six months in liquid nitrogen in vials of 10 million cells until use.

CD14+ monocytes were plated at 150,000 cells/cm^2^, either in 48-well plates for TRAP staining or in 24-well plates for RNA extraction. The media consisted of αMEM, 1% P/S, 5% fetal bovine serum supplemented with recombinant human Macrophage Colony-Stimulating Factor (rhMCSF, Miltenyi Biotec) at 25 ng/mL. This media was changed at day 2 and 5, and supplemented with 20% conditioned media from MSC culture and either of 100 ng/mL recombinant human Receptor Activator of Nuclear factor Kappa-B Ligand (rhRANK-L, Miltenyi Biotec) for osteoclast differentiation or, recombinant human Granulocyte–Macrophage Stimulating Factor and Interleukin-4 (rhGM-CSF & rhIL-4, R&D Systems, both 50 ng/mL) for multinucleated giant cell formation. Neutralizing antibodies were also added at the media change, at 5 µg/mL (human CXCR-1, human CXCR-2 and human gp130 antibodies, R&D Systems). The non-interference of a control IgG2A with basal osteoclast differentiation was confirmed on one donor. After 8 days, cells were either fixed in formalin for 20 min for TRAP staining or lysed for RNA extraction.

### Viability and apoptosis assays

The LIVE/DEAD viability kit for mammalian cells (Invitrogen, L3224) was used to visualize the status of cells on the biomaterial and in spheroids. The samples were washed 3 times with PBS and incubated in culture conditions for 30 min in a PBS solution of the two reagents (calcein-AM for living cells and ethidium homodimer-1 for dead cells) diluted 2000 times. The staining solution was replaced by PBS and images were taken within 30 min.

Metabolic activity was measured with a resazurin assay (Sigma-Aldrich, R7017). A 2 mM solution was prepared and diluted at 0.2 mM in culture media. The preparation was incubated 3 h with the cells in culture conditions and the end-point fluorescence was measured on a microplate reader (Berthold). For Crystal Violet staining, cells were fixed for 5 min by addition of glutaraldehyde to the culture media (1% final concentration). Fixed cells were washed twice in distilled water and stained for 5 min with a solution of Crystal Violet (Sigma-Aldrich, HT901) at 0.1% in 20% ethanol. After several washes in water, plates were let to dry before image acquisition.

Apoptosis was evaluated by measuring caspases activity with the kit Apo-ONE Homogeneous Caspase-3/7 Assay (Promega, G7791). Proteins were extracted in RIPA buffer from attached cells and potential cells in the supernatant, retrieved by centrifugation. Samples were incubated at room temperature overnight with the kit’s reagent diluted at 1/100 in the buffer before fluorescence measurement. Proteins were dosed with a bicinchoninic acid assay and bovine serum albumin was used as a standard.

### TRAP Staining

After fixation, cells were stored up to 2 weeks in PBS at 4 °C or immediately stained. The wells were incubated for 30 min at 37 °C in a buffer containing 40 mM Sodium Acetate, 10 mM Sodium Tartrate at pH = 5. The buffer was removed and a 1:1 mixture of acetone and 100% ethanol was applied to the cell layer for 30 s. The samples were left to dry for 2 min before being incubated in a staining solution in buffer of 0.6 mg/mL Fast Red Violet LB salt (Sigma-Aldrich, 3381) and 100 µL/mL of a solution at 10 mg/mL Naphthol-AS-MX phosphate (Sigma-Aldrich, N4875) in N,N-dimethylformamide (Sigma-Aldrich, 227056) for 30 min. Wells were then washed with distilled water and dried for image acquisition.

### RT-qPCR

All steps followed the manufacturer’s recommendations. RNA was extracted using the NucleoSpin RNA Plus kit (Macherey-Nagel, 740984.250). Reverse transcription was performed with the Maxima H Minus First Strand cDNA Synthesis Kit (Thermo Scientific, K1652). Real time PCR was carried out on a CFX96 (Bio-Rad) with SYBR Select Master Mix (Applied Biosystems, 4472920) with *HPRT* as a reference gene and using the primers in Table 1.

**Table 1:** List of primers used for RT-qPCR experiments.

Target	Primer forward	Primer reverse
*ACP5* (TRAP)	AAGACTCACTGGGTGGCTTTG	GGCAGTCATGGGAGTTCAGG
*BMP2*	AGGACCTGGGGAGCAGCAA	GCTCTTTCAATGGACGTGTCCC
*CALCR*	CCCTTTGCTTCTATTGAGCTG	AAGAATTGGGGTTGGGTGAT
*CD68*	GAACCCCAACAAAACCAAG	GATGAGAGGCAGCAAGATG
*CTSK*	GCCAGACAACAGATTTCCATC	CAGAGCAAAGCTCACCACAG
*CXCR1*	GCAGCTCCTACTGTTGGACA	ATCCCACATCTGTGGATCTGT
*CXCR2*	GGCACAGTGAAGACATCGGT	TTAAATCCTGACTGGGTCGCTG
*DCSTAMP*	TGCATGCAAAGCTGCTTAAA	AGGACTGGAAGCCAGAAATG
*IL6ST* (gp130)	GGACCAAAGATGCCTCAACT	CTTGGACAGTGAATGAAGATCG
*CCL2* (MCP-1)	GCAATCAATGCCCCAGTCAC	TCTTGAAGATCACAGCTTCTTTGG
*CSF1* (M-CSF)	GTTTGTAGACCAGGAACAGTTGAA	CGCATGGTGTCCTCCATTAT
*CXCL1 (*GROα)	AATTCACCCCAAGAACATC	CTGTTCAGCATCTTTTCGAT
*CXCL2* (GROβ)	GAACATCCAAAGTGTGAAGG	GATTTGCCATTTTTCAGC
*CXCL3* (GROγ)	TCAAGAACATCCAAAGTGTG	GCTCCCCTTGTTCAGTATC
*CXCL8* (IL-8)	CATACTCCAAACCTTTCCAC	TCAAAAACTTCTCCACAACC
*CXCL12* (SDF-1)	CCAAACTGTGCCCTTCAGAT	TGGCTGTTGTGCTTACTTGTTT
*HPRT1*	TGACCTTGATTTATTTTGCATACC	CGAGCAAGACGTTCAGTCCT
*IL1B*	CCGGGACTCACAGCAAAA	GGACATGGAGAACACCACTTG
*IL6*	TCCACAAGCGCCTTCGGTCCAG	CTCAGGGCTGAGATGCCGTCG
*IL10*	GCCTTGTCTGAGATGATCC	ACTCATGGCTTTGTAGATGC
*ITGAV*	ATTCTGTGGCTGTCGGAGAT	CCTTGCTGCTCTTGGAACTC
*LIF*	ACGCCACCTGTGCCATACGC	GCTCCCCCTGGGCTGTGTAATAGAG
*MARCO*	TCCCTAGCTGTGGTGGTCAT	CGCCTGCAGATTCAGAACTT
*MMP9*	GAACCAATCTCACCGACAG	GCCCCAGAGATTTCGACTC
*NFATC1*	GGTCTTCGGGAGAGGAGAAA	TGACGTTGGAGGATGCATAG
*OSM*	AGTACCGCGTGCTCCTTG	CCCTGCAGTGCTCTCTCAGT
*TGFB1*	GAGCCCAAGGGCTACCAT	GGGTTATGCTGGTTGTACAGG
*TNF*	CAGCCTCTTCTCCTTCCTGAT	GCCAGAGGGCTGATTAGAGA
*TNFSF11* (RANK-L)	TCGTTGGATCACAGCACATCA	TATGGGAACCAGATGGGATGTC
*TNFRSF11A* (RANK)	CAGATGCCCACAGAAGATGAATAC	CCAGGCTCAGTGAGGAACAG
*TNFRSF11B* (OPG)	CAGCTCACAAGAACAGACTTTCC	TCGAAGGTGAGGTTAGCATGTC
*VEGFA*	CCTTGCTGCTCTACCTCCAC	CCACTTCGTGATGATTCTGC

### BioPlex Assay

For immunoassay detection of cytokine levels in the CM (UNT and STS) from five MSC donors, the Human XL Cytokine Discovery Base Kit (R&D Systems LUXLM000) was used on a Bio-Plex® 200 system (Bio-Rad), following the manufacturer’s recommendations.

### MS-based quantitative proteomics

UNT-CMs and STS-CMs from three MSC donors were produced as previously described and concentrated 20-fold (from 20 to 1 mL) in Amicon Ultra-15 3kD filters (Millipore). The final protein concentration was estimated by the bicinchoninic acid assay and samples were stored at -20 °C, diluted in 1X Laemli buffer (from 4X stock solution). The proteins from each sample were stacked in a single band in the top of a SDS-PAGE gel (4–12% NuPAGE, Life Technologies) and stained with Coomassie blue R-250 (Bio-Rad) before in-gel digestion using modified trypsin (Promega, sequencing grade) as previously described^29^. The resulting peptides were analyzed by online nanoliquid chromatography coupled to tandem MS (Ultimate 3000 RSLCnano and Q-Exactive HF, Thermo Scientific). Peptides were sampled on a 300 μm × 5 mm PepMap C18 precolumn (Thermo Scientific) and separated on a 75 μm × 250 mm C18 column (Reprosil-Pur 120 C18-AQ, 1.9 μm, Dr. Maisch) using a 240-min gradient. MS and MS/MS data were acquired using the Xcalibur software (Thermo Scientific).

Peptides and proteins were identified using Mascot (version 2.6.0, Matrix Science) through concomitant searches against Uniprot database (Homo sapiens taxonomy, September 2019 version), homemade classical contaminant database and the corresponding reversed databases. Trypsin/P was chosen as the enzyme and two missed cleavages were allowed. Precursor and fragment mass error tolerances were set at respectively at 10 and 25 mmu. Peptide modifications allowed during the search were: Carbamidomethyl (C, fixed), Acetyl (Protein N-term, variable) and Oxidation (M, variable). The Proline software^30^ was used to filter the results: conservation of rank 1 peptides, peptide-spectrum-match score ≥ 25, peptide length ≥ 7 amino acids, false discovery rate of peptide-spectrum-match identifications < 1% as calculated on peptide-spectrum-match scores by employing the reverse database strategy, and minimum of 1 specific peptide per identified protein group. Proline was then used to perform a compilation, grouping and MS1 quantification of the protein groups based on specific peptides.

Statistical analysis was performed using ProStaR^31^. Proteins identified in the reverse and contaminant databases, and proteins exhibiting less than three abundance values in one condition were discarded from the list. After log2 transformation, abundance values were normalized by median centering before missing value imputation (slsa algorithm for partially observed values in the condition and DetQuantile algorithm for totally absent values in the condition). Statistical testing was conducted using limma test. Differentially expressed proteins were sorted out using a log2 (fold change) cut-off of 1 and a p-value cut-off of 0.03, allowing to reach a FDR inferior to 5% according to the Benjamini–Hochberg procedure.

Proteins found differentially abundant between UNT-CM and STS-CM were submitted to functional classification using PANTHER v.15.0^32^. Their enrichment in Reactome pathways was tested using Fisher’s exact test. Enrichment was considered for a Bonferroni adjusted *p* value < 0.05.

### Statistical analysis

Data representation and statistical analysis were performed on GraphPad Prism software 6.0. Paired t-test was used to compare two groups of matching data. For comparison of three groups and more, repeated measure one- or two-way ANOVA was carried out, followed by Tukey’s multiple comparison test. Differences were considered significant for *p* value < 0.05 (*), very significant for *p* value < 0.01 (**) and extremely significant for *p* value < 0.001 (***) and *p* value < 0.0001 (****).

## Results

### MSCs’ secretome on BCP is pro-osteoclastogenic

**Figure 1:**
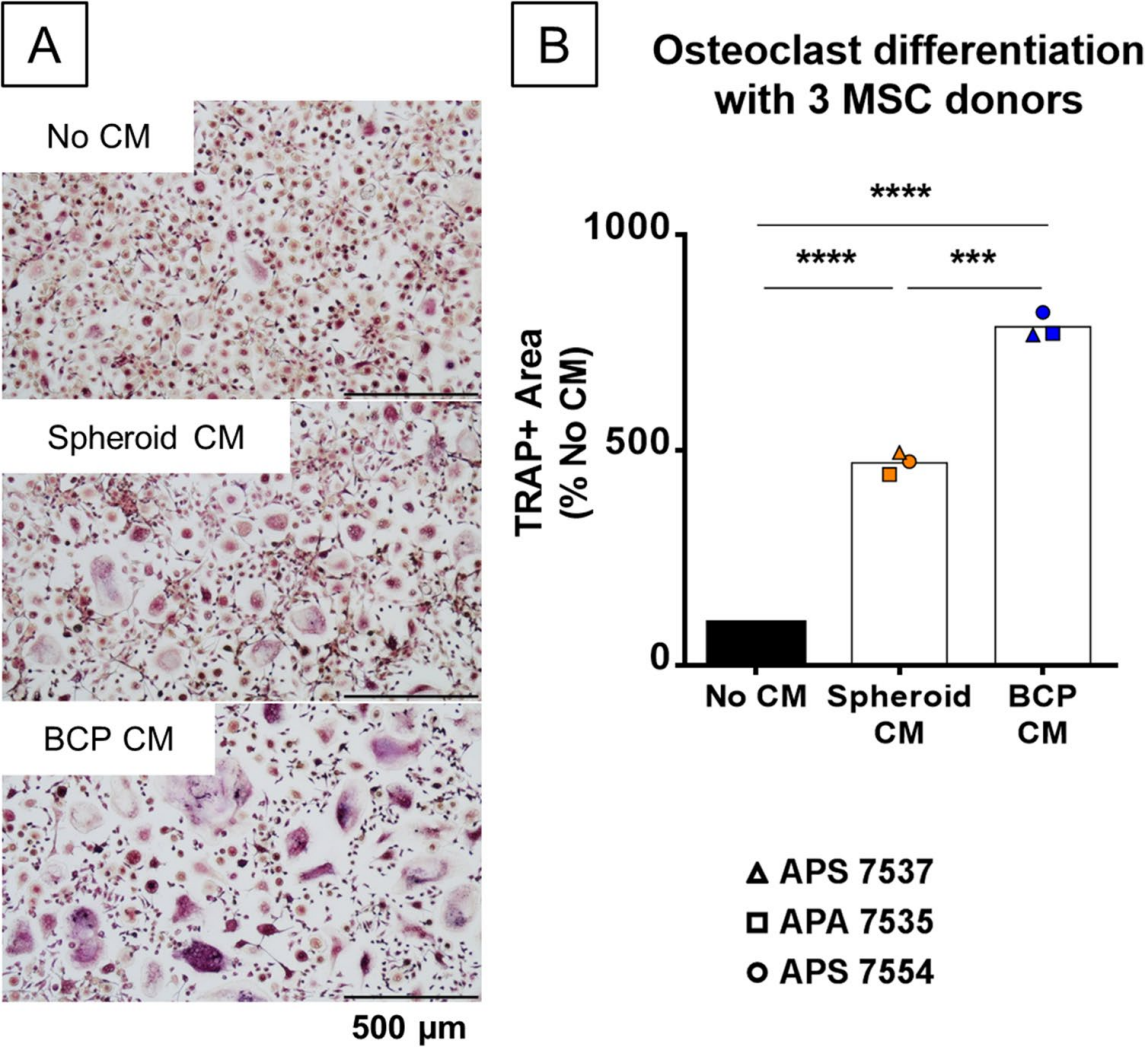
Osteoclastogenesis is enhanced in vitro by CM from MSCs culture on BCP material. (**A**) Microscopic images of TRAP-stained osteoclasts culture without CM, with CM from MSC spheroids (Spheroid CM) or MSCs on BCP material (BCP CM). (**B**) Quantification by image analysis of TRAP+ area after 8 days of osteoclast differentiation with spheroid or BCP CM from three MSC donors, normalized to the TRAP+ area without CM. N = 3 independent experiments with technical triplicate. Statistical analysis by repeated measure ANOVA followed by Tukey’s multiple comparison test, *** for *p* value < 0.001, **** for *p* value < 0.0001.

In order to study the secretions of MSCs in contact with the biomaterial, short duration (48 h) cell cultures were performed before recovering the CM. To mimic in vivo implantation from previous studies^1^, a large number of cells (400,000) were seeded on biomaterial granules (50 mg) in a small volume (500 µL) of serum-free media. The use of MSC spheroids as a control allowed us to preserve the cell/volume ratio, essential as the CM is later used in an osteoclast culture. The CMs were centrifuged to avoid adding dead cells or debris to the osteoclast differentiation test. Osteoclasts were differentiated from human CD14+ monocytes stimulated with recombinant human M-CSF and RANK-L. As shown in Fig. 1A, addition of CM from MSC spheroid cultures significantly increased the number and size of osteoclasts. CM from MSC/BCP cultures had an even stronger effect leading to the formation of huge and strongly TRAP+ multinucleated cells and this effect was consistent with three different MSC donors (Fig. 1B). We choose to focus on what made the seeding on BCP so efficient for osteoclast stimulation as this mechanism may come into play for in vivo implantation, even though the CM from the spheroid culture also improved osteoclast differentiation compared to the baseline. Additionally, increasing the exogenous RANK-L concentration two to four times did not change the size and shape of osteoclasts formed (data not shown), ensuring saturating conditions in this major cytokine. CM obtained on BCP granules without cells did not have any effect on osteoclasts either (data not shown), confirming that MSC-secreted factors other than RANK-L were responsible for the phenotypical changes in osteoclasts.

## MSCs grown in vitro on BCP are undergoing apoptosis

**Figure 2:**
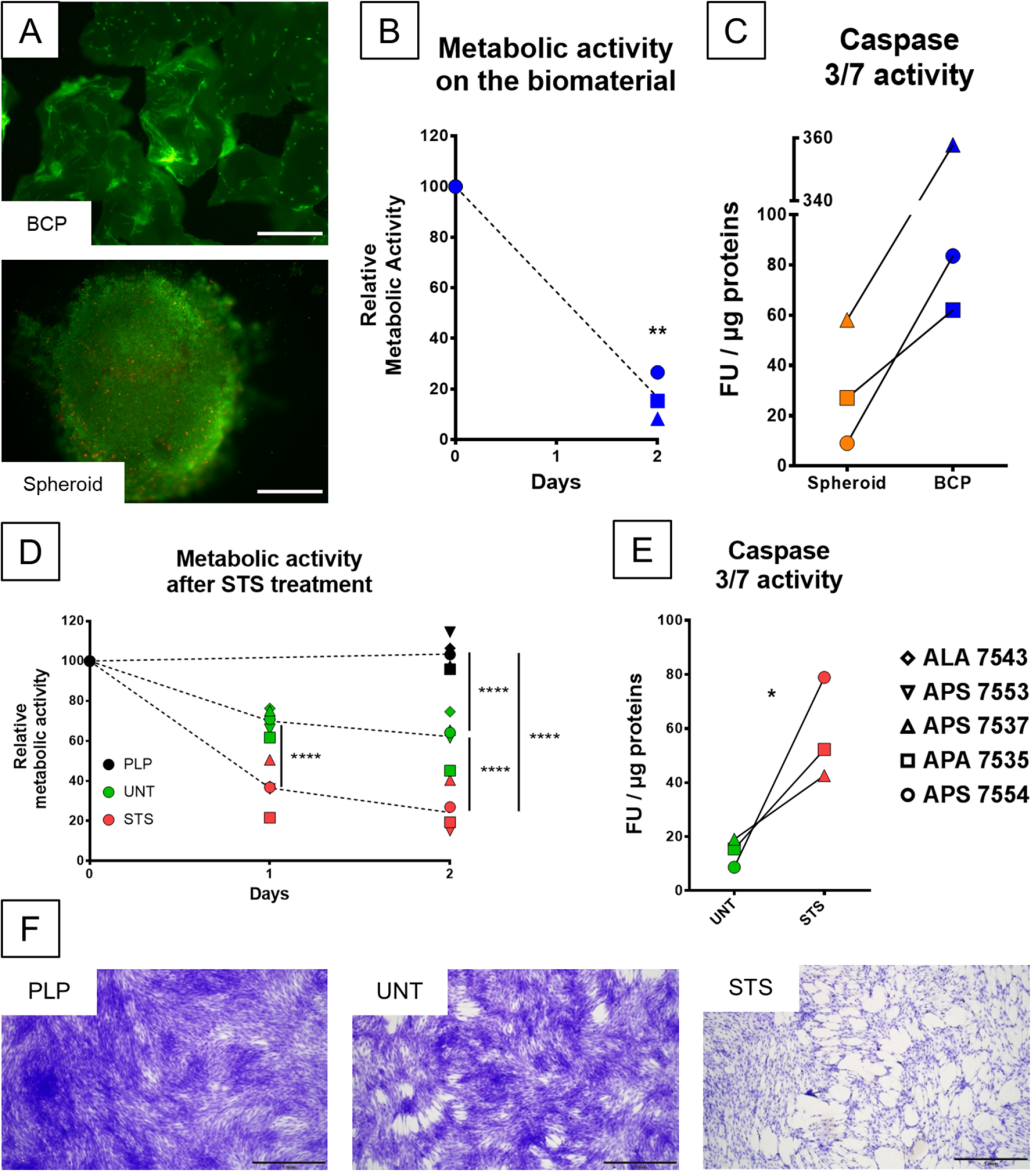
MSCs seeded on BCP material undergo apoptosis, reproduced in 2D by a staurosporine treatment. (**A**) LIVE/DEAD® staining at 48 h of MSCs seeded on BCP particles or in spheroid form. (**B**) Viability measure by resazurin assay after 48 h of MSCs from three donors on the BCP material, normalized to the value after seeding. N = 3 independent experiments with technical triplicate. Statistical analysis by one-sided paired t-test, ** for *p* value < 0.01 (**C**) Caspase 3/7 activity per µg of proteins extracted from spheroid or BCP cultures of three MSC donors. N = 3 independent experiments with technical triplicate. (**D**) Viability measure by resazurin assay after 24 and 48 h of MSCs from five donors, in complete media (PLP) in serum-free media (untreated, UNT) or in serum-free media after a 4 h 0.1 µM STS treatment (STS), normalized to the value before treatment. N = 5 independent experiments with technical triplicate. Statistical analysis by repeated measure ANOVA followed by Tukey’s multiple comparison test, **** for *p* value < 0.0001. (**E**) Caspase 3/7 activity per µg of proteins extracted from untreated (UNT) or STS-treated (STS) cultures of three MSC donors. N = 3 independent experiments with technical triplicate. Statistical analysis by one-sided paired t-test, * for *p* value < 0.05. (F) Crystal violet staining of MSCs after 48 h in complete media (PLP), serum-free media (UNT) or serum-free media after STS-treatment (STS).

After two days of culture, Live/Dead staining (Fig. 2A) showed living MSCs attached on the biomaterial surface and almost no dead cells, however cell density appeared lower than what would be expected according to the quantity of cells seeded. Dead cells may have been removed with the collected media or washed away during the staining protocol. In the MSC spheroid, cells were visible as a cohesive mass, entrapped by the secreted matrix, and only sporadic dead cells were observed. As shown in Fig. 2B, 48 h after seeding, the metabolic activity measured from MSCs on BCP dropped to around 20% of its initial value, corroborating the fluorescent images. The metabolic activity of the cells could only be recorded on the MSC/BCP culture, as the reagent did not diffuse efficiently inside the MSC spheroids, indicating that the MSC spheroid culture may not be a good control in this instance as it might also prevent the diffusion of soluble mediators into the CM. In line with these results, it was found that caspase 3/7 activity was higher although not significantly in cell lysate from cells grown on BCP compared to spheroids prepared from the three MSC donors, suggesting an increased apoptosis for MSCs grown on the biomaterial (Fig. 2C).

To further investigate the implications of MSCs undergoing apoptosis and to simplify the model, MSCs in a classical 2D culture were subjected to a staurosporine (STS) treatment, artificially inducing cell death by apoptosis. STS is an inhibitor of protein kinases, widely used for in vitro induction of apoptosis by activation of caspase-3-like proteases^33^. A mild treatment with 0.1 µM STS during 4 h in serum-free conditions caused a loss of metabolic activity in MSCs from five human donors of 60% at 24 h and 80% at 48 h (Fig. 2D). In parallel, culture of the same cells in serum-free conditions without STS also exhibited a diminished metabolic activity, less drastic compared to treated cells, averaging 75% and 60% of their initial value at 24 and 48 h, respectively. In contrast, in cells grown with complete culture media containing platelet lysate, metabolic activity was maintained after 2 days (Fig. 2D). Importantly, MSCs treated with STS showed a significant increase, of two to eight-folds, in caspase 3/7 activity per µg of proteins compared to untreated cells, confirming their death by apoptosis (Fig. 2E). A crystal violet staining allowed visualization of healthy cell morphologies in complete or serum-free media while revealing rounded cells and cell layer disruption after STS treatment (Fig. 2F). These data indicated that STS treatment mirrored the apoptotic stress induced by seeding MSCs in large numbers on BCP materials and could facilitate the production of CM in more controlled conditions.

### Supernatants from apoptotic MSCs favor osteoclasts but inhibit MNGCs

**Figure 3:**
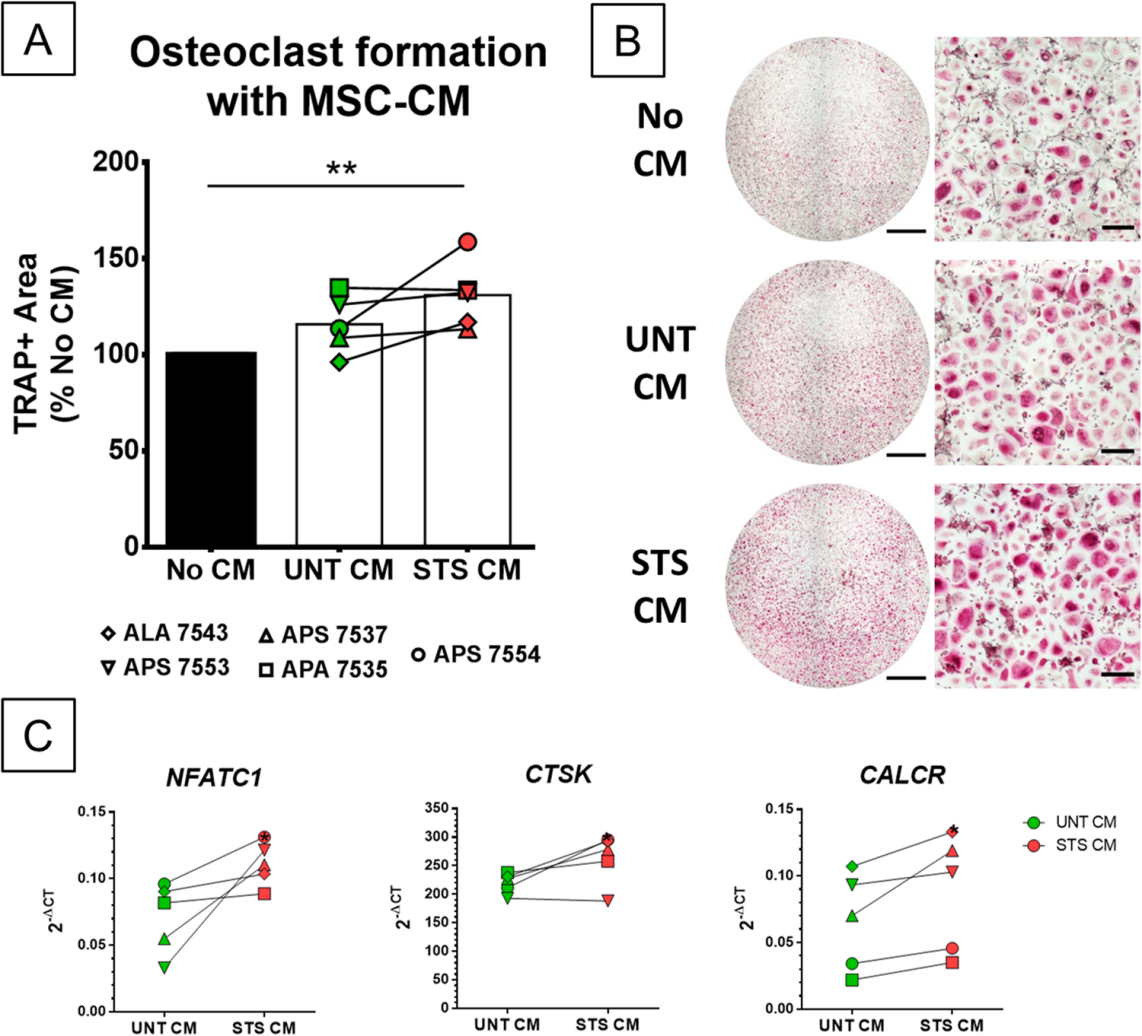
CM from STS-treated MSCs exhibit pro-osteoclastogenic properties. (**A**) Quantification by image analysis of TRAP+ area after 8 days of osteoclast differentiation with UNT or STS CM from five MSC donors with two or three CD14+ donors, normalized to the TRAP+ area without CM. N = 5 values averaged from 2 or 3 independent experiments with different CD14+ cells with technical triplicate. Statistical analysis by repeated measure ANOVA followed by Tukey’s multiple comparison test, ** for *p* value < 0.01 (**B**) Full well (scale bar = 2 mm) and close-up (scale bar = 200 µm) images of TRAP-stained osteoclasts at 8 days of differentiation without CM, with UNT-CM and STS-CM. (C) Comparative expression of differentiation markers in osteoclasts treated with UNT or STS-CM from five MSC donors. N = 5 independent experiments with technical triplicate. Statistical analysis by one-sided paired t-test, * for *p* value < 0.05.

CM of untreated MSCs (UNT-CM) and STS-treated MSCs (STS-CM) were used in osteoclasts and MNGCs cultures. Results obtained from five MSCs donors and two to three CD14+ monocytes donors indicated that STS-CMs significantly favor osteoclastogenesis in comparison to cultures without CM but not significantly compared to UNT-CM (Fig. 3A). In comparison to UNT-CM, two STS-CMs (donors APS 7554 and ALA 7543) strongly stimulated osteoclastogenesis, whereas two only slightly induced it (donors APS 7553 and APS 7537) and one did not (APA 7535). The number of donors was too small to analyze this variability further but CMs obtained with MSCs from donor APS 7554 consistently showed a significant stronger pro-osteoclastogenic effect after STS treatment, using CD14+ monocytes from three different donors (Fig. 3B). To confirm the superior effect of STS-CMs compared to UNT-CM on osteoclastogenesis, the expression of specific genes was evaluated using RT-qPCR in osteoclasts stimulated with the CM from the five MSCs donors. A significant over-expression of three markers of osteoclast differentiation was observed with STS-CMs compared to UNT-CM (Fig. 3C): cathepsin K (*CTSK*), nuclear factor of activated T cells 1 (*NFATC1*) and the calcitonin receptor (*CALCR*). The expression of other osteoclast markers, such as dendrocyte expressed seven transmembrane protein (*DCSTAMP*) and matrix metallopeptidase 9 (*MMP9*), was not modulated between the two conditions (data not shown). With the MSC donor APS 7554, gene expression analysis (Figure S1) confirmed the consistent overexpression of classical markers of differentiation (*NFATC1*, *CTSK*, *CALCR* & *ACP5/TRAP*) in osteoclasts grown with STS-CM compared to osteoclasts cultured in absence of CM. STS-CM treated osteoclasts also had a modulated inflammatory phenotype with increased expression of *TNFRSF11B/OPG* and *IL-8/CXCL-8* but reduced *TNF*, *IL1B* and *IL10*.

**Figure 4:**
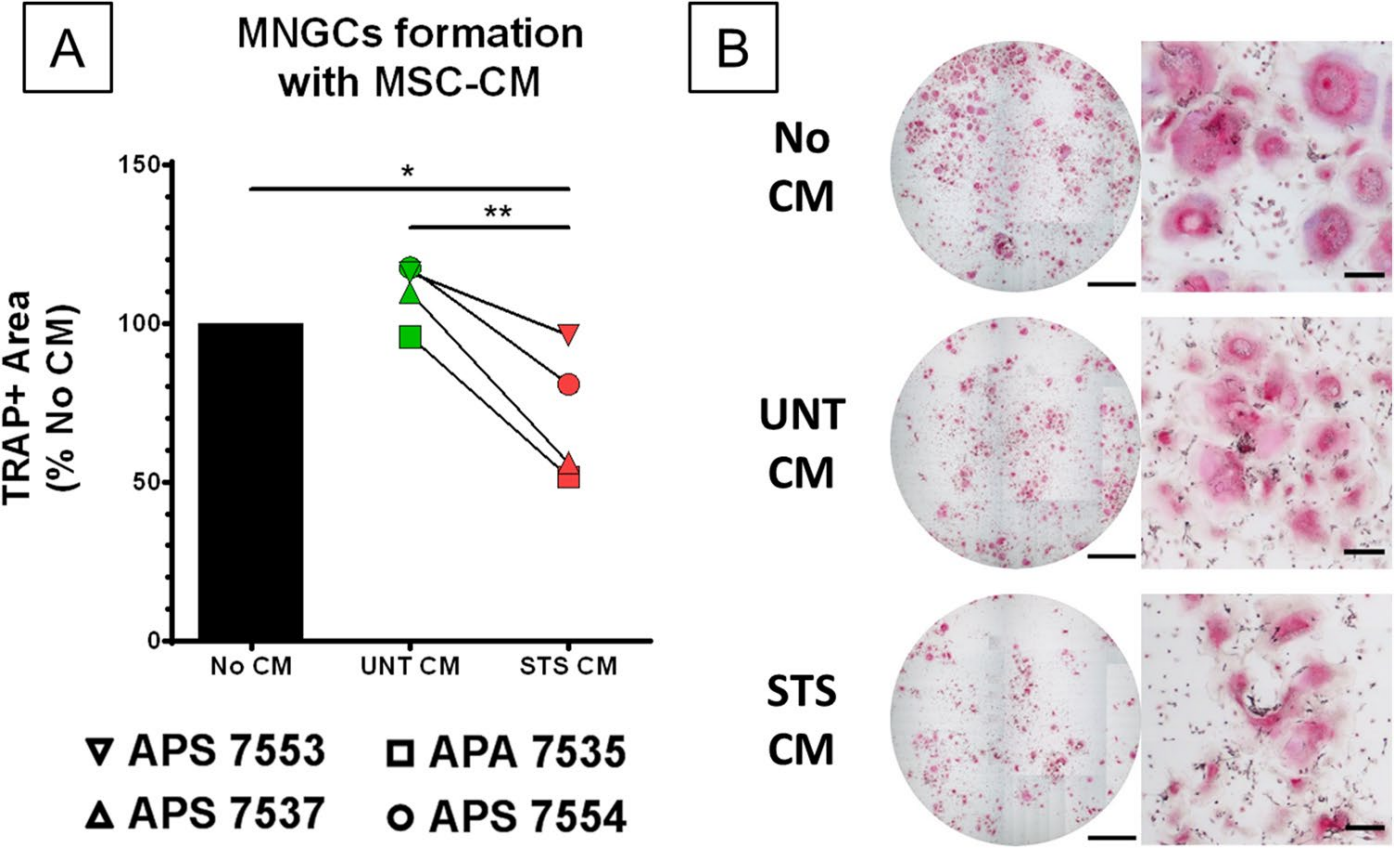
CM from STS-treated MSCs inhibit the development of GM-CSF/IL-4 induced MNGCs. (**A**) Quantification by image analysis of TRAP+ area after 8 days of MNGC differentiation with UNT or STS CM from four MSC donors, normalized to the TRAP+ area without CM. N = 4 independent experiments with technical triplicate. Statistical analysis by repeated measure ANOVA followed by Tukey’s multiple comparison test, * for *p* value < 0.05, ** for *p* value < 0.01. (**B**) Full well (scale bar = 2 mm) and close-up (scale bar = 200 µm) images of TRAP-stained MNGCs at 8 days of differentiation without CM, with UNT-CM and STS-CM.

We then tested the impact of STS-CM on MNGC formation. For this, MNGCs culture conditions were validated based on the literature (GM-CSF & IL-4, both 50 ng/mL)^34^ and followed the same timing as used for osteoclast differentiation. MNGCs formation was assessed with the same staining protocol used for osteoclasts as they also express the TRAP enzyme. DAPI/Phalloidin staining confirmed their multinucleation and a brief comparison with osteoclasts’ gene expression profile revealed low expression of osteoclast-specific markers (*NFATC1*, *CTSK*, *CALCR*, *MMP9*) but higher expression of cytokines such as *IL-6*, *IL-1B* or *IL-10* and of the protein essential for cell fusion DC-Stamp (Figure S2). Using four MSCs donors, we showed that STS-CM significantly inhibited the formation of MNGCs compared to UNT-CM (Fig. 4). Therefore, CMs from MSC cultures after induction of apoptosis have opposite effects on CD14+ monocytes fate; inhibiting MNGC differentiation of CD14+ monocytes stimulated with GM-CSF and IL-4 while promoting osteoclast differentiation with M-CSF and RANK-L.

## MSCs undergoing apoptosis have an altered secretion profile

**Figure 5:**
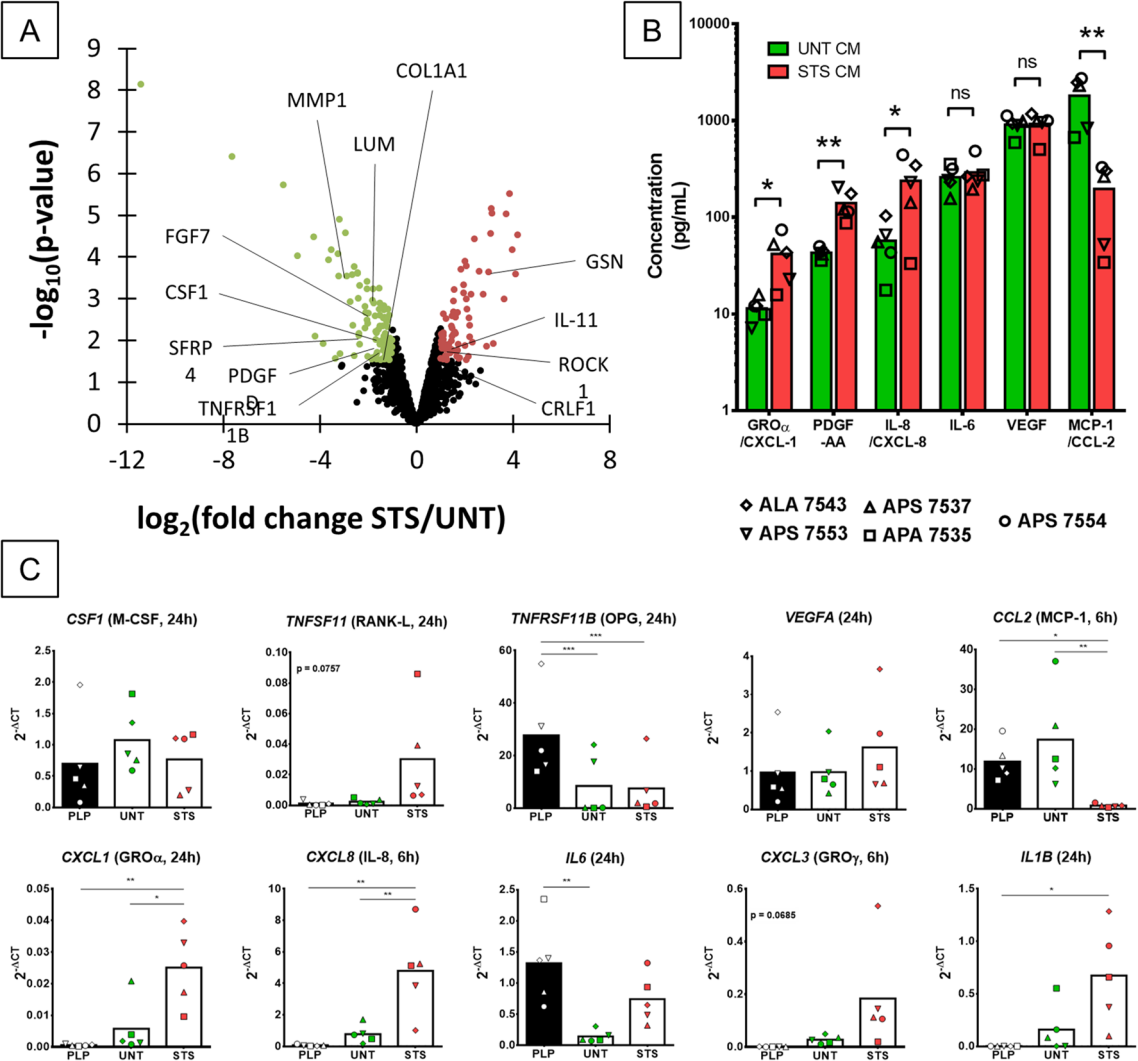
Soluble factors present in the media drastically change upon STS treatment. (**A**) Volcano plot displaying the differential abundance of proteins detected in STS-CM and UNT-CM by MS-based proteomics. The volcano plot represents the -log10 (p-value) on y axis plotted against the log2 (Fold Change STS/UNT) on x axis. Red and green dots represent proteins found more abundant respectively in STS-CM and UNT-CM (*p* value ≤ 0.03 and fold change ≥ 2). (**B**) Quantification of cytokines concentration detected by multiplex immunoassay in UNT and STS-CM from 5 MSC donors. N = 5 independent experiments with technical duplicate. Statistical analysis by one-sided paired t-test, ns = not significant, * for *p* value < 0.05, ** for *p* value < 0.01. (**C**) Gene expression analysis in MSCs from five donors in complete media (PLP) or after 6 and 24 h in serum-free media, with (STS) or without (UNT) STS-treatment. N = 5 independent experiments. Statistical analysis by repeated measure ANOVA followed by Tukey’s multiple comparison test, * for *p* value < 0.05, ** for *p* value < 0.01, *** for *p* value < 0.001.

To better characterize the secretion profile of MSCs, we performed high-throughput proteomic analysis using MS-based analysis and multiplex immunoassay. MS-based quantitative analyses of the proteins contained in CM from 3 MSC donors in UNT or STS conditions led to the identification and quantification in the three replicates of one condition of 1420 proteins. Statistical analysis highlighted 181 proteins with a differential abundance, 76 being more abundant in STS-CM and 105 being more abundant in UNT-CM (fold change ≥ 2 and *p* value ≤ 0.03, Fig. 5A, Data S1). Among the classical soluble mediators (cytokines, chemokines and growth factors), 2 cytokines of the IL-6 family were enriched in STS-CM (CRLF1 and IL-11), 13 mediators were unchanged (such as TGFB1, BMP-1, IGF2, CXCL-12 or IL-6) and 5 were downregulated (such as PDGF-D, M-CSF or OPG) (Table 2). Bioinformatic analyses (Data S2) emphasized that STS-CMs were enriched in apoptosis-linked pathways, notably through proteins that are associated to the cytoskeleton (GSN, ROCK1), and in the interleukin-12 signaling pathway (e.g. CRLF1). On the contrary, UNT-CM enriched proteins were linked to the extracellular matrix, with components such as collagens (e.g. COL1A1) or proteoglycans (e.g. LUM), and its degradation (e.g. MMP1). These findings were consistent with a classical MSC phenotype in the untreated condition while STS treatment induced an apoptotic stress.

**Table 2:** Major soluble mediators differentially present in STS-CM compare to UNT-CM.

Upregulated	Unchanged	Downregulated
*CRLF1* *IL-11* **bFGF** **GRO/CXCL-1** **GRO/CXCL-2** **IL-8/CXCL-8** **PDGF-AA**	*ADIPOQ* *LIF* *TGFB1* *TGFB2* *PDGF-C* *BMP-1* *NOTCH3* *GDF6* *MIF* *IGF2* *INHBA* *SDF-1/CXCL-12* ***IL-6*** **RANTES** **VEGF**	*SFRP4* *FGF7* *PDGF-D* *CSF-1/M-CSF* *OPG/TNFRSF11B* **Eotaxin/CCL-11** **MCP-1/CCL-2**

Proteins detected by LC–MS (italics), Bioplex experiment (bold) or both (bolditalics).

In addition, the CMs from 5 MSC donors were analyzed by a multiplex immunoassay towards 45 cytokines. Among the targeted proteins, 22 were detected in at least one sample but only 9 with a value above the lowest standard point (Table S1). Figure 5B present the six well-detected proteins for which a statistical analysis could be conducted. Three were statistically enriched in STS-CM; GROα/CXCL-1 with only 3 values above the lowest standard point but always in STS-CMs (3.5-fold increase, *p* < 0.05), IL-8/CXCL-8 (fourfold increase, *p* < 0.05) and PDGF-AA (threefold increase, *p* < 0.01). The only protein detected in both the immunoassay and MS-based analysis was IL-6, whose concentration did not change between the UNT and STS conditions. VEGF concentration was also found to be equivalent between UNT- and STS-CMs. MCP-1/CCL-2 was the only cytokine depleted in STS-CM (ninefold decrease, *p* < 0.01). Other cytokines were only detected at low levels (close or below the lowest standard point), thus conclusions on their regulation should be drawn with caution. GROβ/CXCL-2 and basic Fibroblast Growth Factor (bFGF) were only detected in some STS-CM, suggesting an increased production or secretion during apoptosis. Eotaxin/CCL-11 was found downregulated in STS-CM but with only one value above the lowest standard point. All measurements for FMS-like tyrosine kinase 3 ligand (FLT3L) were below the lowest standard but it could be enriched in STS-CM (Table S1).

The expression of detected cytokines as well as other potential soluble modulators of osteoclastogenesis, such as M-CSF, RANK-L and osteoprotegerin (OPG), was evaluated by RT-qPCR at 6 and 24 h after STS treatment (Fig. 5C and data not shown). *TNFSF11/RANKL* transcript seemed to be upregulated after STS treatment compared to either the untreated condition (serum-free media) or the basal condition in complete media, but the difference was not statistically significant, most probably because of a great variability between the replicates. M-CSF and OPG proteins were both reported as downregulated in STS-CM compared to UNT-CM by MS-base quantitative proteomics, but the expression of their transcript was found similar with or without treatment. These results suggest that the increased abundance of these proteins in UNT-CM compared to STS-CM is linked either to differential post-transcriptional regulations or enhanced secretion in UNT-CM. *TNFRSF11B/OPG* mRNA expression was however lower in serum-free conditions (UNT and STS) than in complete media. The RANK-L/OPG ratio could overall be more favorable to osteoclasts after STS treatment. Overexpression of GROα/CXCL-1, IL-8/CXCL-8 and downregulation of MCP-1/CCL-2 were confirmed at the transcriptional level. Similarly, *VEGFA* expression was unchanged in all tested conditions. *IL6* expression, stable after 6 h, significantly dropped in serum-free untreated condition at 24 h. Expression of *PDGFA* was below the detection limit and expression of *CXCL2* was detected at low level in only some STS-treated MSCs (data not shown). In addition, screening of other mediators revealed a potential overexpression of *CXCL3* (*p* = 0.0685) and a significant one of *IL1B* (Fig. 5C).

Overall, radical changes of MSC secretions occur after STS treatment. The proteomic analysis illustrate that apoptotic cells lost their normal secretion profile while releasing intracellular components. Several mediators enriched in STS-CM could influence osteoclasts development.

### Blocking CXCR-1/CXCR-2 abrogates the effect of MSC-CM but also alters basal osteoclastogenesis

**Figure 6:**
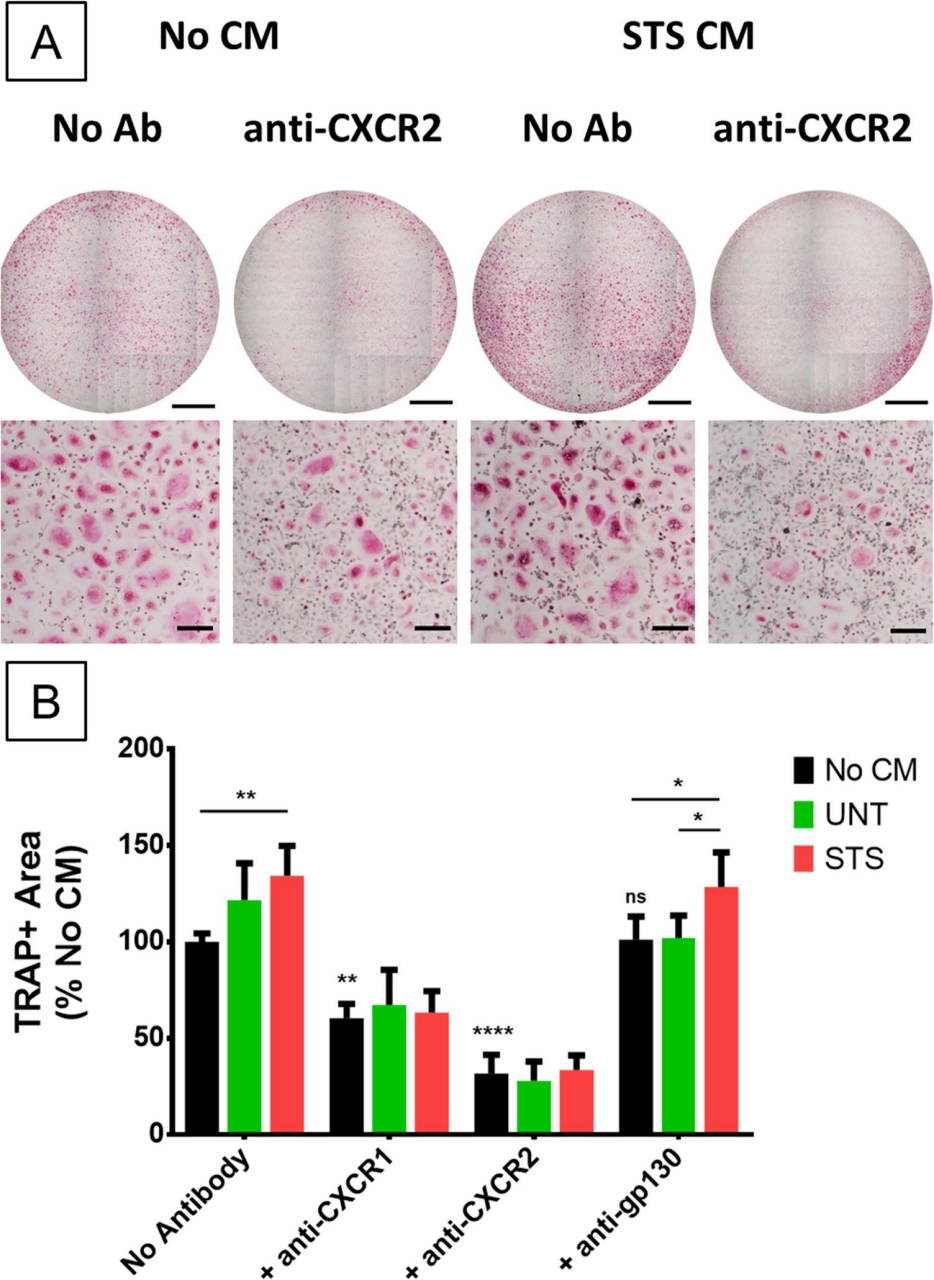
MSC-mediated induction of osteoclastogenesis is alleviated by an anti-CXCR1 or anti-CXCR2 antibody but not anti-gp130. (**A**) Full well (scale bar = 2 mm) and close-up (scale bar = 200 µm) images of TRAP-stained osteoclasts at 8 days of differentiation with or without STS-CM and with or without anti-CXCR-2 antibody. (**B**) Quantification by image analysis of TRAP+ area after 8 days of osteoclast differentiation with UNT or STS-CM from MSC of donor APS 7554, with or without neutralizing antibodies towards CXCR-1, CXCR-2 or gp130, normalized to the TRAP+ area without CM and antibody. N = 3 technical replicates, representative experiment out of two. Error bars represent the standard deviation to the mean value. Statistical analysis by repeated measure ANOVA followed by Tukey’s multiple comparison test, comparison between the conditions with the same antibody or with the control “No CM, No antibody”, * for *p* value < 0.05, ** for *p* value < 0.01, **** for *p* value < 0.0001.

To gain a deeper understanding of the cytokines involved in the communication between MSCs and osteoclasts, neutralizing antibodies were employed. For this experiment, CMs from the donor giving the most marked and reproducible results were used (APS 7554). Given the combined results of proteomics, multiplex analysis (Table 2) and RT-qPCR, and the literature on osteoclasts regulation, CXCR-1 and CXCR-2 were targets of choice as receptors for IL-8 and CXCL-1 to -3. The effect of IL-6 on osteoclasts is controversial^35^ but since it is one of the most abundant cytokines and it shares the gp130 receptor with other detected cytokines such as LIF or IL-11, the effect of an anti-gp130 antibody was evaluated. An analysis by RT-qPCR confirmed that osteoclasts expressed the 3 receptors CXCR-1, CXCR-2 and gp130 (Figure S1). As previously observed in Fig. 3, STS-CM obtained with MSC donor APS 7554 significantly increased the TRAP area compared to the basal culture (Fig. 6). Addition of an anti-CXCR-1 or anti-CXCR-2 but not anti-gp130 antibody erased the difference induced by STS-CM. Antibodies targeting CXCR-1 and -2 also impacted basal osteoclasts differentiation. Blocking CXCR-2 had an even more significant effect, possibly due to its ligands not utilizing CXCR-1, such as CXCL-1, -2 and -3. Consequently, GROα/CXCL-1 and IL-8/CXCL-8 seemed implicated in the activation of osteoclastogenesis as ligands of these two receptors upregulated in STS-CM. Given the impact of the antibodies on basal osteoclast differentiation, complementary experiments blocking the expression of these cytokines in MSCs would strictly confirm their implication.

## Discussion

This study demonstrated that MSCs seeded on a BCP material had a strong pro-osteoclastic effect. Similarly, to an in vivo implantation, few cells survived on the biomaterial compared to the number seeded. Perhaps due to their high seeding concentration or to the interaction with the material, most cells went through apoptosis, as detected by caspases activity. This apoptosis could be replicated in a 2D model by STS treatment. The conditioned media from STS-treated culture retained this pro-osteoclastic characteristic, although significantly diminished in comparison to CM of MSCs seeded on the biomaterial. Conversely, they inhibited the formation of IL-4 stimulated MNGCs. A deep proteomic analysis could be performed, completed by a specific cytokine detection assay, to determine the major players of these communications between MSCs and myeloid cells. It was found that GROα/CXCL-1, GROβ/CXCL-2 and IL-8/CXCL-8 were significantly enriched in STS-CM, as well as *CXCL3* (GROγ) mRNA expression in STS-treated MSCs. Blocking them with specific antibodies against their receptors CXCR-1 or -2 supported their pivotal role in osteoclastogenesis, although additional experiment should be performed. The RANK-L/OPG ratio was potentially increased after STS treatment. However, RANK-L is unlikely to be the major mediator of osteoclast stimulation in our assay as exogenous RANK-L concentrations were saturating. Also, it is primarily a transmembrane, cell surface associated cytokine and could be essential in vivo when MSCs are in direct contact with macrophages participating in the inflammatory reaction towards the biomaterial. Overall, we provide here first evidences that several CXCL chemokines could have a key role in the crosstalk between apoptotic MSCs and osteoclasts.

The major mediators of MSCs’ stimulatory effect towards osteoclastogenesis seemed to be IL-8/CXCL-8 and other CXCR-2 ligands (CXCL-1, -2 and -3). This is supported by previous in vitro studies reporting positive effects of these CXCL chemokines on monocytes migration and osteoclasts differentiation^36–38^. Given the complexity of MSCs’ secretome, these proteins may not be the only ones involved, and additional studies are warranted to identify additional players. Incidentally, there are most likely positive and negative regulators of osteoclastogenesis co-secreted by MSCs and neutralizing antibodies could therefore unbalance this equilibrium. This would explain why blockage of one of the two receptors for IL-8 was sufficient to suppress the stimulatory effect of the CM. In addition, osteoclasts self-stimulation, mostly based on IL-8/CXCL-8, is essential during osteoclastogenesis^39^ and seem to be activated by STS-CM. The observed increase in *CXCL8/IL8* expression in osteoclast culture with STS-CM could either be due to a higher differentiation rate or to a specific improvement of autocrine signaling by MSCs’ secretome through a different pathway. Also, IL-8 has been reported as an enhancer of bone regeneration by MSC recruitment^40^ and could have a key role in regeneration beyond osteoclast differentiation. Interestingly, CMs produced from apoptotic normal dermal human fibroblast (NHDF) cultures seemed to also activate osteoclastogenesis (Figure S3). This effect was also associated with an IL-8 burst.

One of the limitations of this work is that it does not inform on the potential second step of the mechanism, where osteoclasts would recruit new osteoblast progenitor and locally favor bone formation on the biomaterial. The phenotype of osteoclasts formed in presence of STS-CM needs to be further investigated. In Figure S1, we present preliminary data revealing differential expression of several cytokines in STS-CM treated osteoclasts. These changes could be involved in the complex processes allowing the transition from early inflammation to bone formation. A lower expression of *TNF* and *IL1B* is in line with a less inflammatory phenotype but it is associated with a reduced *IL10* expression, a major anti-inflammatory cytokine. Also, increased *TNFRSF11B/OPG* expression should inhibit osteoclasts, but higher *CXCL8/IL8* production could participate in their self-induction. Levels of these cytokines need to be more precisely measured at the protein level, which was complicated here due to the presence of MSC-CM. In addition, chemoattractant for skeletal stem cells and coupling factors should be measured as potential key proteins to revive a bone remodeling-like cycle.

It is important to keep in mind that this communication with osteoclasts is not the only role of MSCs. It is well established that they have a strong immunomodulatory potential. Their crosstalk with macrophages, for example, has been particularly studied for bone regeneration applications^41^. The effect of MSCs’ secretome during apoptosis on immune cells involved in early inflammation (macrophages, neutrophils or mast cells) should also be investigated to improve our understanding of the process of bone induction by MSC-CaP as a whole. Here, we limited our observations to the effect of artificial apoptosis, but other parameters may participate to cell death or modulate the activity of implanted MSCs. Hypoxia, for example, was previously reported to impact MSCs’ secretion profile, notably inducing the expression of IL-8 among other mediators^42^. The biomaterial is crucial in the interaction with host cells through mechanotransduction but its composition^43^ and surface properties^44^ also influence MSCs phenotype. Since MSCs could have a perivascular origin^45,46^, one may relate this osteoinduction mechanism to fracture healing where blood vessels are disrupted after trauma releasing many MSCs in the microenvironment. These cells may then undergo apoptosis due to the lack of oxygen and nutriments and lead to osteoclasts differentiation rather than MNGCs, although the balance is tight.

## Conclusion

These results showed that secretions from apoptotic MSCs globally favored osteoclastogenesis and inhibited formation of MNGCs in vitro. The effect on osteoclasts seems linked to IL-8 and other CXCL chemokines, ligands of the receptors CXCR-1 and -2, but future studies are needed to decipher the complete mechanism of action of these chemokines in the crosstalk between MSC and osteoclasts. The mediators responsible for the effect on MNGCs remain to be determined. Here, we link for the first time the two main observations of preclinical experiments using MSCs for bone regeneration, i.e. MSCs clearance by apoptosis from the implantation site and osteoclasts formation preceding bone formation. Apoptosis is a key event in natural tissue regeneration through apoptosis-induced proliferation^47,48^. In bone regeneration, osteoclasts’ apoptotic bodies improved defect bridging in mice^49^ as well as osteogenic differentiation in vitro^50^. In the treatment of graft-versus-host disease, MSCs immunosuppressive function has been tightly linked to their apoptosis^51^. These observations support the hypothesis putting osteoclasts in the center of bone formation induced by MSC-CaP constructs and further question the relationship between osteoclasts and MNGCs. These insights into the mechanism of MSC-based bone regeneration may constitute an additional step towards cell free approaches^52^.

## Supplementary Information

Supplementary Figure S1.

Supplementary Figure S2.

Supplementary Figure S3.

Supplementary Information 1.

Supplementary Information 2.

Supplementary Table S1.
