# Supplementary figures and images for: Apoptotic mesenchymal stromal cells support osteoclastogenesis while inhibiting multinucleated giant cells formation in vitro
Source: Sci Rep. 2021 Jun 9;11:12144. doi: 10.1038/s41598-021-91258-4 (PMC8190145; doi:10.1038/s41598-021-91258-4)

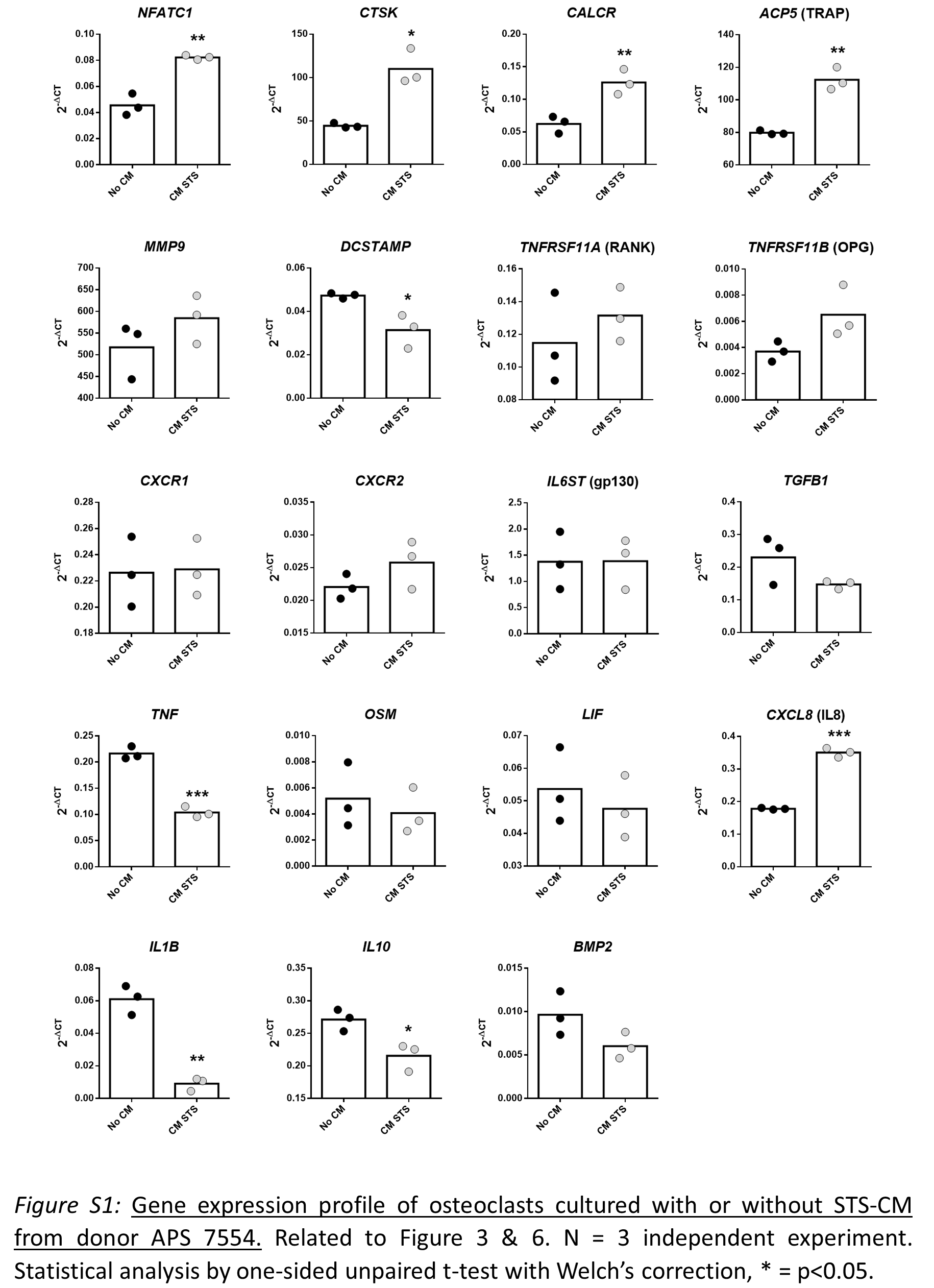

Supplement: Supplementary file 1 — Supplementary Figure S1. [file 41598_2021_91258_MOESM1_ESM.tif]

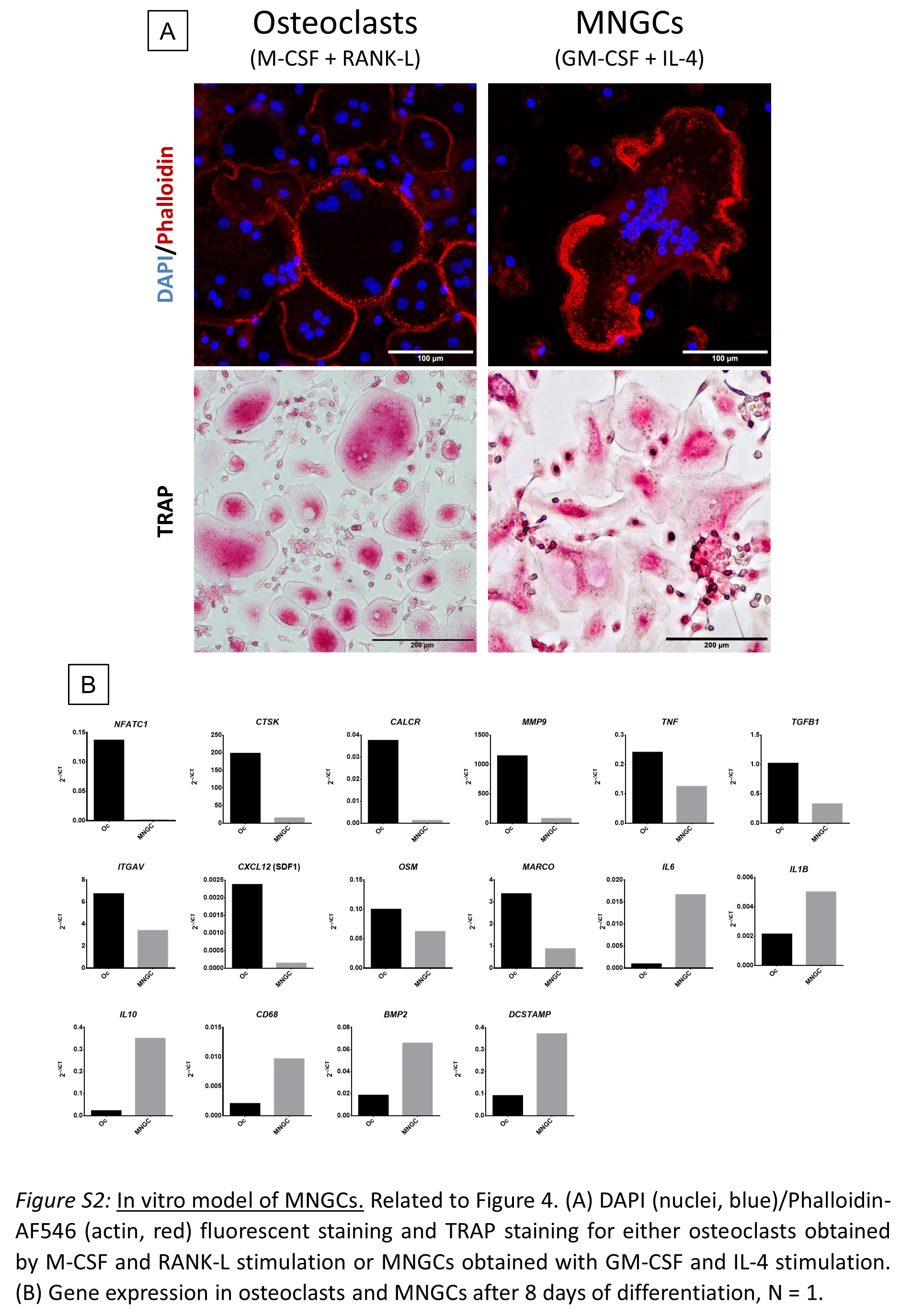

Supplement: Supplementary file 2 — Supplementary Figure S2. [file 41598_2021_91258_MOESM2_ESM.tif]

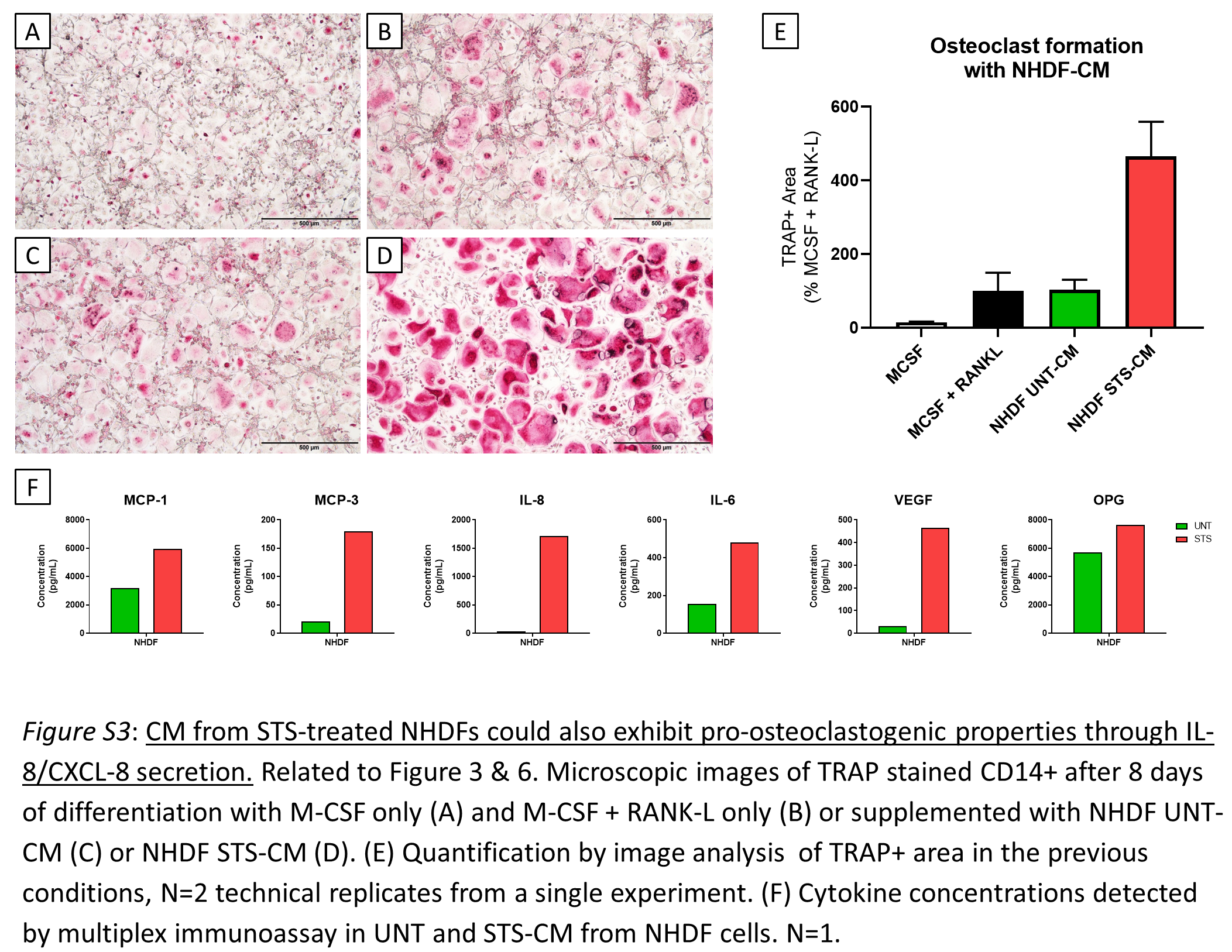

Supplement: Supplementary file 3 — Supplementary Figure S3. [file 41598_2021_91258_MOESM3_ESM.tif]
