# Supplementary material for: Apoptotic mesenchymal stromal cells support osteoclastogenesis while inhibiting multinucleated giant cells formation in vitro
Source: Sci Rep. 2021 Jun 9;11:12144. doi: 10.1038/s41598-021-91258-4 (PMC8190145; doi:10.1038/s41598-021-91258-4)
Supplement: Supplementary file 6 — Supplementary Table S1. [file 41598_2021_91258_MOESM6_ESM.docx]

|  |  | **Eotaxin** | **FGF basic** | **Flt3 ligand** | **Fractalikine** | **GM-CSF** | **Granzyme B** | **GROa** | **GROb** | **IFN-a** | **IL-1ra** | **IL-2** |
| --- | --- | --- | --- | --- | --- | --- | --- | --- | --- | --- | --- | --- |
| **APS 7535** | NT | *10.80 | OOR < | OOR < | *52.40 | OOR < | *1.25 | *9.86 | OOR < | *0.64 | *0.34 | OOR < |
|  | STS | *10.01 | *11.09 | OOR < | *52.40 | OOR < | *1.01 | *15.74 | OOR < | *0.50 | *0.84 | OOR < |
| **37** | NT | *20.24 | OOR < | OOR < | *65.67 | *1.01 | *1.51 | *15.74 | OOR < | *1.53 | *1.35 | OOR < |
|  | STS | *11.58 | 21,17 | *6.45 | *60.23 | OOR < | *1.51 | 52,33 | *10.80 | *1.53 | *7.30 | *0.66 |
| **54** | NT | 44,02 | OOR < | *2.54 | *65.67 | *1.01 | *1.51 | *12.07 | OOR < | *1.38 | *1.35 | OOR < |
|  | STS | *10.01 | *17.86 | *29.34 | *68.45 | OOR < | *1.51 | 74,22 | 25,35 | OOR < | *2.92 | *1.90 |
| **43** | NT | *40.73 | OOR < | *6.45 | *65.67 | *0.20 | *1.25 | *12.07 | OOR < | *0.93 | *0.84 | *0.21 |
|  | STS | *11.58 | OOR < | *24.73 | *65.67 | OOR < | *1.51 | 43,28 | 24,71 | *0.93 | *2.92 | *1.83 |
| **53** | NT | *13.06 | OOR < | OOR < | *60.23 | OOR < | *1.25 | *7.16 | OOR < | *0.50 | *0.84 | OOR < |
|  | STS | *10.01 | OOR < | *10.04 | *64.30 | OOR < | *1.25 | *22.83 | OOR < | *0.50 | *0.34 | *0.80 |
|  |  | **IL-4** | **IL-6** | **IL-8** | **IL-15** | **MCP-1** | **MIP-1a** | **MIP-3a** | **PDGF-AA** | **PDGF-AB/BB** | **RANTES** | **VEGF** |
| **35** | NT | OOR < | 353,24 | 17,61 | OOR < | 668,35 | OOR < | OOR < | 35,81 | *0.35 | *109.17 | 592,73 |
|  | STS | *0.02 | 273,53 | 33,16 | OOR < | 34,15 | OOR < | OOR < | 87,01 | *0.25 | *149.04 | 503,64 |
| **37** | NT | OOR < | 155,95 | 55,5 | OOR < | 2309,5 | *4.33 | OOR < | 41,59 | *1.03 | *261.91 | 980,46 |
|  | STS | *0.22 | 194,68 | 141,74 | *0.26 | 264,18 | *4.33 | OOR < | 120,98 | *0.94 | *187.58 | 987,43 |
| **54** | NT | OOR < | 316,8 | 43,16 | OOR < | 2729,89 | *3.67 | OOR < | 49,84 | *0.94 | *187.58 | 1121,25 |
|  | STS | OOR < | 484,08 | 442,14 | *0.36 | 328,43 | OOR < | *0.21 | 114,34 | *0.04 | *187.58 | 1000,03 |
| **43** | NT | OOR < | 231,81 | 103,66 | OOR < | 2479,47 | OOR < | OOR < | 44,89 | *0.84 | *187.58 | 939,16 |
|  | STS | *0.11 | 263,94 | 344,68 | *0.26 | 302,39 | *1.85 | OOR < | 175,12 | *0.45 | *149.04 | 1172,85 |
| **53** | NT | OOR < | 241,23 | 65,63 | OOR < | 836,32 | OOR < | OOR < | 42,42 | *0.35 | *109.17 | 892,37 |
|  | STS | OOR < | 237,82 | 229,57 | *0.07 | 52,32 | OOR < | OOR < | 204,42 | *0.04 | *67.30 | 958,69 |

*Table S1*: Concentrations in pg/mL of the 22 cytokines detected by multiplex immunoassay in at least one sample of UNT or STS-CM from five MSC donors. * indicates a value below the lowest standard point and OOR< indicates an Out Of Range low value.
